# The Safety and Immunogenicity of a 13-Valent Pneumococcal Polysaccharide Conjugate Vaccine (CRM197/TT) in 12–23-Month Children: A Double-Blind, Randomized, Phase III Trial in China

**DOI:** 10.3390/vaccines13121190

**Published:** 2025-11-24

**Authors:** Zhiqiang Xie, Feiyu Wang, Lili Huang, Haitao Huang, Guangwei Feng, Xue Wang, Jiebing Tan, Xiaomin Ma, Wangyang You, Xiaolong Li, Jinbo Gou, Yanxia Wang

**Affiliations:** 1 Henan Province Center for Disease Control and Prevention, Zhengzhou 450003, China; xiezqshang@163.com (Z.X.); 13643826177@163.com (L.H.); vacfeng@163.com (G.F.); tanjiebingz@163.com (J.T.); dsrt12345@163.com (W.Y.); 2 CanSino Biologics Inc., Tianjin 300457, China; feiyu.wang@cansinotech.com (F.W.); haitao.huang@cansinotech.com (H.H.); xue.wang@cansinotech.com (X.W.); xiaomin.ma@cansinotech.com (X.M.); xiaolong.li@cansinotech.com (X.L.)

**Keywords:** 13-valent pneumococcal conjugate vaccine, CRM197/TT, immunogenicity, safety

## Abstract

**Objectives**: This study systematically assessed the safety and immunogenicity of a new 13-valent pneumococcal conjugate vaccine (CRM197/TT, PCV13i) against the licensed PCV13 vaccine in a cohort of Chinese children between 12 and 23 months of age. **Methods**: This is a phase III, randomized, double-blind trial (NCT04841369). A total of 528 participants were randomized 1:1 to receive two doses of either PCV13i experimental or the PCV13 control vaccine at a 2-month interval, with 517 participants completing the vaccinations. **Results**: The overall incidence of adverse events (37.12% vs. 32.70%, *p* = 0.134) and adverse reactions (24.81% vs. 21.61%, *p* = 0.221) was comparable between the experimental and control groups. Local adverse reactions were more frequent in the experimental group (10.00% vs. 6.12%, *p* = 0.021), such as erythema (7.88% vs. 4.02%, *p* = 0.008). Systemic adverse reactions, including fever (10.77% vs. 13.77%), showed no significant differences. No vaccine-related serious adverse events occurred. Immunogenicity assessments showed that seropositivity rates for most serotypes reached ≥96% in both groups, with eight serotypes achieving 100% seropositivity in the experimental group. PCV13i induced higher IgG geometric mean concentrations (GMCs) for serotypes 3, 7F, and 19F (*p* < 0.05), whereas the control group showed higher GMCs for serotypes 1, 5, 6A, and 14 (*p* < 0.05). Opsonophagocytic activity (OPA)-related geometric mean titers (GMTs) were superior for PCV13i against serotypes 7F (39,583 vs. 17,249, *p* < 0.001) and 19F (1517 vs. 983, *p* = 0.028), but lower for serotype 5 (13 vs. 93, *p* < 0.001). **Conclusions**: PCV13i demonstrated non-inferior immunogenicity and an acceptable safety profile in 12–23-month-old children.

## 1. Introduction

*Streptococcus pneumoniae* was included as one of the 12 prior pathogens due to its high carriage rates, genetic adaptability and ability to shift from a commensal to a pathogenic interaction with its host [1,2]. According to research, 27–65% of children and <10% of adults are carriers of *S. pneumoniae* [3]. In addition. *S. pneumoniae* has developed resistance to multiple antibiotics including penicillin, macrolides, fluoroquinolone and sulfamethoxazole-trimethoprim [4,5]. Invasive pneumococcal disease (IPD) is a serious condition caused by infection with *Streptococcus pneumoniae* bacteria, often leading to invasive manifestations such as bacteremia, meningitis, and pneumonia with bacteremia [6,7]. IPD remains a significant global health concern, particularly among young children, the elderly, and immunocompromised individuals [8,9].

The burden of IPD is particularly pronounced in children under two years of age, with high rates of morbidity and mortality observed in this vulnerable population [10,11]. In a 2015 active population-based surveillance of hospitalized community-acquired pneumonia in adult patients in the US, pneumococcus constituted 5% of the documented cases [12]. An average of 1.2 million young children worldwide dies due to pneumococcal pneumonia and meningitis. In developed countries, the mortality rate for pneumococcal pneumonia can reach 11–40%. In addition, a study showed trends in overall rate of IPD in different age groups between the years 1998–2016 in the United States. PCV13 has reduced the incidence of IPD in young children by 36%. In 2006, it was reported that between 23 and 30% of children still carried pneumococci with no evidence of a decline in carriage prevalence [13]. In China, approximately 70% of patients <5 years of age diagnosed with IPD at a single children’s hospital during 2010–2017 were 1–<5 years of age, and IPD hospitalization rates were 11.2 and 13.3 per 100,000 among children 1–<2 and 2–<5 years of age, respectively [14]. In the Canary Islands, there was a 60.1% decrease in the incidence of IPD due to PCV13 serotypes in vaccinated children (from 13.1 to 4.4 cases per 100,000 children between 2001 and 2010). A similar situation was observed in other places such as Navarra, with reductions in pediatric IPD from 39 cases per 100,000 children in the period 2008–2010 to 18 cases in 2011–2014, with an overall 90% reduction in the rate of IPD caused by all serotypes and 95% vaccine effectiveness in IPD due to PCV13 serotypes [15].

The World Health Organization (WHO) has suggested that PCV13 should be administered in multiple doses starting at 2 months of age, with subsequent doses at 4, 6, and 12–15 months. For unvaccinated children aged 12–23 months, they should receive 2 doses at an interval of at least 2 months [16]. However, in Chinese national immunization programs, pneumococcal vaccines against pneumococcal infections, particularly conjugated vaccines targeting prevalent serotypes, which can significantly reduce the incidence of IPD in vaccinated populations, were not be integrated [17,18,19]. Pneumococcal polysaccharide conjugate vaccine (CRM197/TT) (PCV13i) employs both CRM197 and TT carrier proteins to potentially enhance immune responses across serotypes. When a single vector protein is overloaded, it causes vector-specific B cells to monopolize limited T helper cell resources, thereby suppressing the immune response to polysaccharide haptenes. the PCV13i dual-carrier protein system effectively establishes two independent T-cell-mediated pathways. This alleviates the risk of carrier-induced epitope suppression (CIES) associated with single-carrier overload, ensuring immune responses to each polysaccharide are not competitively inhibited. Consequently, the dual-carrier strategy delivers balanced and potent immunogenicity across all included serotypes, overcoming the fundamental limitations of high-valent single-carrier vaccines [20,21]. Previously, we published a clinical trial study results of the safety and immunogenicity of PCV13i in 2-month age and the results showed PCV13i was well tolerant and immunogenic in infants [22]. Here, we would like to report the safety and immunogenicity of PCV13i in 12–23-month Chinese children.

## 2. Methods

### 2.1. Study Plan and Participants

This study was a Phase III, randomized, double-blind clinical trial that unfolded across three sites in China’s Henan Province from April 2021 to September 2022 (NCT04841369, ClinicalTrials.gov). The study enrolled eligible children aged 12–23 months who had not previously received any pneumococcal vaccine. The main exclusion criteria include premature labor (delivery before 37 weeks of gestation) with low birth weight (female < 2300 g, male < 2500 g); history of obstructed labor, birth asphyxia, or nervous system injury; pre-vaccination axillary temperature > 37.0 °C; history of epilepsy, seizure, or convulsions, or a family history of psychosis; known or suspected severe conditions affecting the respiratory, cardiovascular, hepatic, renal, or integumentary systems, as well as malignancies; and acute exacerbations of any chronic diseases; other situations not suitable for this clinical trial as judged by the investigator. If participants had serious anaphylactic reactions or newly identified or emerging conditions after receiving the first dose of vaccines, they may be excluded for second doses.

The trial enrolled 528 participants, who were randomly allocated at a 1:1 ratio to receive either the investigational PCV13i vaccine or the licensed PCV13 vaccine. Each participant received two doses of their assigned vaccine, with the two administrations separated by a two-month interval. The study protocol and informed consent forms received approval from the Ethics Committee of the Henan Provincial Center for Disease Control and Prevention (Approval No.: 2018-YM-006-02) prior to trial initiation. The trial was carried out in accordance with the “Good Clinical Practice” of the National Medical Products Administration, and Declaration of Helsinki. Written informed consent has been obtained before screening.

### 2.2. Vaccines and Masking

All vaccinations were delivered as a 0.5 mL intramuscular injection into the upper arm’s deltoid muscle. The investigational product, PCV13i (CanSino Biologics Co., Ltd., Tianjin, China. lot: PCV202009002C), contains polysaccharides from 13 pneumococcal serotypes (1, 3, 4, 5, 6A, 6B, 7F, 9V, 14, 18C, 19A, 19F, 23F). These polysaccharides are fermented and purified before being conjugated to either CRM197 or tetanus toxoid (TT) carrier proteins. The final formulation is adsorbed onto an aluminum phosphate adjuvant. The comparator was the commercially available PCV13 (Pfizer, New York, NY, USA. lot: DK0537DK2065).

The randomization process was performed using Stata 16/SE software, which facilitated the 1:1 allocation of participants to either the experimental or control vaccine group. In order to maintain blindness in the trial, the mapping between the alphabetic code corresponding to the subject number and the alphabetic code corresponding to the vaccine number is known only to the unblinded researchers, and is sealed after the assignment is completed. None of the other investigators involved in the clinical trial knew the mapping between the subject number and the vaccine number.

### 2.3. Immunogenicity Assessment

A standardized opsonophagocytic assay (OPA) was used to quantify the ability of serum antibodies to mediate the opsonization and subsequent phagocytic killing of *Streptococcus pneumoniae*. Prior to analysis, serum samples were heat-inactivated to eliminate nonspecific complement activity. Differentiated HL-60 cells were employed as phagocytes and prepared by centrifugation, washing with 1 × HBSS, and resuspension in opsonophagocytosis assay buffer (OBB), followed by viable cell counting. Bacterial working stocks of relevant serotypes were rapidly thawed, washed, and resuspended in OBB to the appropriate concentration. Serially diluted serum samples (3-fold dilutions) were incubated with the bacterial suspension for 30 min at room temperature under 700 rpm agitation to facilitate opsonization. Subsequently, a mixture of HL-60 cells and complement (4:1 ratio) was added to each well, with heat-inactivated complement controls included. The plate was incubated for 45 min at 37 °C under 5% CO_2_ at 700 rpm. The reaction was terminated by transferring the plate to ice. A 10 μL aliquot from each well was plated onto THYA plates and overlaid with antibiotic-containing soft agar. After 16–18 h of incubation at 37 °C with 5% CO_2_, bacterial colonies were enumerated using an automated colony counter.

### 2.4. Outcomes

The primary immunogenicity endpoints were defined as the seropositivity rate (antibody concentration ≥ 0.35 μg/mL, WHO recommended [23]) and the geometric mean concentration (GMC) of serotype-specific IgG antibodies, measured 30 days following the two-dose vaccination series. The primary safety endpoint was the incidence of adverse reactions within 30 days after vaccination. Secondary endpoints encompassed the geometric mean titer (GMT) and the seropositivity rate (titer ≥ 1:8) of opsonophagocytic activity (OPA) antibodies, along with the proportion of subjects achieving serotype-specific IgG antibody concentrations ≥ 1.0 μg/mL at the 30 days post-vaccination.

### 2.5. Statistical Analysis

The sample size calculation was based on the clinical performance of the licensed vaccine, for which a 93% antibody positivity rate was assumed in the control group at 30 days post-vaccination. The calculation employed a one-sided α of 0.025 and a power (1-β) of 90%. Clinical trial results for the control vaccine abroad indicated lower antibody levels for serotypes 3, 4, 5, 6B, 9V, and 23F. To prevent an inflated Type II error for the 6-serotype antibody, the probability of Type II error correction is (1 − 0.90)/6 = 0.0167, with a non-inferiority margin of −0.1 and p0 set at 0.94; The z-test CC is selected for calculating the test statistic. Using these parameters, a sample size calculation was performed in NCSS-PASS software (16.0.1) based on the rate difference for non-inferiority of two independent proportions. This analysis indicated that 238 participants per group were needed for each age cohort. The dropout rate for the 12–23-month-old age group is set at 10%. Considering the blinding factor, the experimental group and control group for the 12–23-month-old age group are planned to enroll 264 participants each.

Safety analysis was based on the safety set (SS). The incidence rate and corresponding severity were calculated, and adverse reaction grading was analyzed based on the number of cases. For immunogenicity analysis, antibody titers were log-transformed, and *t*-tests (when normal and variance aligned) and corrected *t*-tests (when normal but not variance aligned) were used to compare whether there was any difference in antibody GMC before vaccination. To evaluate the robust immune response, the proportion of participants achieving a serotype-specific IgG antibody concentration ≥ 1.0 µg/mL at 30 days post-vaccination was calculated for each group. Inter-group comparisons for these proportions were performed using the χ^2^ test, the corrected χ^2^ test, or Fisher’s exact test, as appropriate. The proportion of serotype-specific OPA titers ≥ 1:8 in the experimental and control groups was statistically analyzed 30 days after vaccination.

## 3. Results

**Figure 1:**
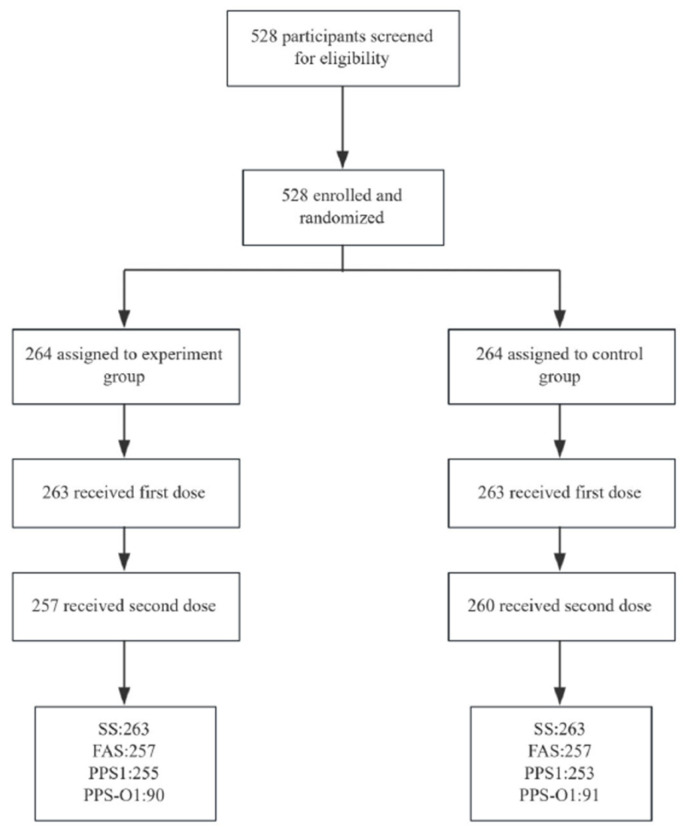
Flow chart. Note: SS: Safety set. All the subjects who received the study vaccines after randomization and had at least one safety evaluation were included in the SS. FAS: Full analysis set. All the subjects who met the inclusion criteria, participated in randomization, received the experimental vaccine, and had the test results of blood samples before immunization or after any immunization were included in the FAS. PPS1: Per Protocol Set. Participants who completed 2 vaccinations with available IgG test results before immunization and after the second immunization. PPS-O1: Participants who can be included in PPS1 (with or without blood collection before the first dose) and have post-immunization OPA test results.

Of the 528 infants initially enrolled and randomly allocated (1:1) to the experimental or control groups, 517 completed the full two-dose vaccination schedule. Immunogenicity assessment was conducted on 514 participants, from whom blood samples were collected 30 days post-second vaccination (Figure 1). The mean age was 18.11 months in the experimental group and 18.05 months in the control group. The gender (*p* = 0.542) and age (*p* = 0.833) compositions were balanced between the experimental and control groups. The two treatment groups demonstrated balanced baseline characteristics, as detailed in Table 1, with no notable differences in age, gender, body mass index (BMI), or pre-vaccination axillary temperature.

### 3.1. Safety

As shown in Table 2, the adverse reactions in the experimental group (n = 520) and the control group (n = 523) were observed and analyzed in detail. For overall adverse events, the incidence rate was 37.12% (193 cases) in the experimental group and 32.70% (171 cases) in the control group, and the difference between the two groups was not statistically significant (*p* = 0.134). In terms of overall adverse reactions, the incidence rate was 24.81% (129 cases) in the experimental group and 21.61% (113 cases) in the control group, with a *p* value of 0.221, which also did not show a significant difference. Regarding local adverse reactions, the incidence of 10.00% (52 cases) in the experimental group was higher than that of 6.12% (32 cases) in the control group and was statistically significant (*p* = 0.021). Further breaking down the local adverse reactions, the incidence of erythema was 7.88% (41 cases) in the experimental group and 4.02% (21 cases) in the control group, with a significant difference of *p* = 0.008; swelling was 3.08% (16 cases) in the experimental group and 2.29% (12 cases) in the control group, with a non-significant difference of *p* = 0.434; pain was 0.38% (2 cases) in the experimental group and 1.15% (1 case) in the control group. The incidence of pain was 0.38% (2 cases) in the experimental group and 1.15% (6 cases) in the control group, *p* = 0.291, the difference was not statistically significant. The incidence of fever was 10.77% (56 cases) in the experimental group and 13.77% (72 cases) in the control group, *p* = 0.140, and the difference between the two groups was not significant. One (0.19%) grade 3 fever occurred in the control group. Irritability occurred in 2 cases (0.38%) in the experimental group and not in the control group, *p* = 0.248. The incidence rate of cough in the experimental group was 2.88% (15 cases) and in the control group was 2.29% (12 cases), *p* = 0.548. The incidence of diarrhea was 2.31% (12 cases) in the experimental group and 1.53% (8 cases) in the control group, *p* = 0.360; the incidence of vomiting was 0.77% (4 cases) in the experimental group and 0.57% (3 cases) in the control group, *p* = 0.994; and the incidence of hypersensitivity was 1 case (0.19%) in the experimental group but not in the control group, *p* = 0.499.

### 3.2. Immunogenicity

Prior to vaccination, seropositivity rates and IgG GMCs for most pneumococcal serotypes were comparable between the experimental and control groups, with the exception of serotypes 18C and 23F. The seropositivity rate was 7.06% in the experimental group and 13.04% in the control group for serotype 18C (*p* = 0.025), and 41.18% in the experimental group and 50.59% in the control group for serotype 23F (*p* = 0.033). After two-dose vaccination, most serotypes showed a significant increase in seropositivity rate of IgG response in both experimental and control groups, even reaching 100%. The seropositive rate of serotypes 1, 4, 7F, 9V, 14, 18C, 19A, 19F in the experimental group and control group reached 100% 30 days post-vaccination, and the rest serotypes reached more than 96%. Among them, serotype 5 was higher in the control group than in the experimental group, and the difference in the seropositive rate between the groups was statistically significant (*p* = 0.007), while the differences between the groups for the remaining serotypes were not statistically significant (*p* > 0.05). The differences between serotypes 3, 7F, and 19F were statistically significant (≥0.05), and the GMC of the experimental group was higher than that of the control group. While the differences between serotypes 1, 5, 6A, and 14 were statistically significant (*p* < 0.05), and the GMC of the control group was higher than that of the experimental group. The differences between serotypes 4, 6B, 9V, 18C, 19A, and 23F between the experimental group and the control group were not statistically significant (Table 3).

Table 4 presented the proportion of participants achieving a serotype-specific pneumococcal IgG antibody concentration ≥ 1.0 μg/mL pre- and post-vaccination in both the experimental and control groups. Before vaccination, the baseline seropositivity rates were low and generally no significant difference was observed between the two groups, except for 19A, where the control group had a higher pre-existing seropositivity (*p* = 0.032). The highest pre-vaccination rates were seen for serotype 14 (86.67% vs. 86.56%) and serotype 19A (50.20% vs. 59.68%). Following vaccination, both groups exhibited a substantial increase in seropositivity rates for all serotypes. Notably, for several serotypes-including 4, 6A, 6B, 7F, 9V, 14, 18C, 19A, and 19F-the post-vaccination rates were very high (exceeding 90% in most cases) and not statistically different between the experimental and control groups (all *p* > 0.05). Seropositivity reached 100% for serotypes 7F, 14, and 19F in both groups. In addition, significant differences in immunogenicity between the groups emerged for three specific serotypes post-vaccination. For serotype 3, the response was significantly higher in the experimental group (57.25%) compared to the control group (37.94%) (*p* < 0.001). For serotype 5, the response was significantly higher in the control group (87.35%) compared to the experimental group (40.00%) (*p* < 0.001). For serotype 23F, the response was significantly higher in the experimental group (95.29%) compared to the control group (87.35%) (*p* = 0.001).

Table 5 showed the percentage of OPA ≥ 1:8 and GMT of 13 different serotypes. In serotype 1, the percentage of OPA ≥ 1:8 in the experimental group was 91.11% and GMT was 31.82, whereas in the control group, the percentage of OPA ≥ 1:8 was 95.60% and GMT was 43.10. In serotypes 3 and 4, the ratio of OPA ≥ 1:8 was 100.00% in both experimental and control groups, with GMT 185.42 in experimental group and 172.48 in control groups for serotype 3, and 2631.87 in experimental group and 2688.68 in control group for serotype 4. The differences in serotype 5 between the experimental and control groups were statistically significant (*p* < 0.001), and both the OPA antibody GMT and percentage was higher in the control group than in the experimental group. In the serotype 6A experimental group, the proportion of OPA ≥ 1:8 was 98.89% with a GMT of 5980.41, compared with 96.70% and 4913.92 in the control group, respectively. The proportion of OPA ≥ 1:8 in the serotype 6B experimental group was 97.78% and GMT was 4135.98, compared with 95.60% and 3046.04 in the control group. The proportion of OPA ≥ 1:8 in the serotype 7F experimental and control groups were 100.00%, with a GMT of 39,583.25 in experimental group and 17,248.59 in control group (*p* < 0.001). The proportions of OPA ≥ 1:8 in both experimental and control groups of serotypes 9V, 14, and 19A were all 100.00%, and the GMTs between experiment group and control group were not significantly different for these three serotypes. The ratio of OPA ≥ 1:8 in the serotype 19F was 96.67% in experimental group and was 98.90% in control group, while the GMT was higher in experimental group (1516.83) compared with that in control group (982.95) (*p* = 0.028).

## 4. Discussion

This study was designed to evaluate the safety and immunogenicity of PCV13i in 12–23-month-old Chinese children. In terms of safety, the overall adverse event incidence was 37.12% in the experimental group and 32.70% in the control group (*p* = 0.134), and the overall adverse reaction incidence was 24.81% and 21.61%, respectively, with no statistically significant differences. However, the incidence of local adverse reactions in the experimental group was higher than that in the control group, and the incidence of erythema was significantly different. The incidence of fever was 10.77% in the experimental group and 13.77% in the control group, with no significant difference, and only one grade 3 fever occurred in the control group. Other adverse reactions such as irritability, cough, diarrhea, vomiting, and hypersensitivity reaction had no statistically significant differences between the two groups. There was no vaccine-related serious adverse event occurred in experimental and control groups. All adverse reactions were occurred in 7 days post-vaccination.

Regarding immunogenicity, before vaccination, except for serotypes 18C and 23F, there were no statistically significant differences in the seropositivity rate and GMC of IgG response between the experimental and control groups for pneumonia serotypes. After vaccination, the seropositivity rate of most serotypes significantly increased in both groups, with some reaching 100%. Specifically, the seropositivity rate of serotype 5 was higher in the control group than in the experimental group. The GMCs of serotypes 3, 7F, and 19F were higher in the experimental group than in the control group (*p* < 0.05), while the GMCs of serotypes 1, 5, 6A, and 14 were higher in the control group than in the experimental group, and there were no statistically significant differences in the GMC of the remaining serotypes between the two groups. For OPA antibodies, the GMT and positive proportion of OPA antibodies of serotype 5 were higher in the control group than in the experimental group, the GMT of serotype 7F was higher in the experimental group than in the control group, and the GMT of serotype 19F was higher in the experimental group than in the control group. The proportion of participants achieving pneumococcal IgG antibody concentration ≥ 1.0 μg/mL illustrated while both vaccines demonstrated strong immunogenicity across most serotypes, the experimental vaccine elicited a significantly superior response for serotypes 3 and 23F, whereas the control vaccine elicited a significantly superior response for serotype 5.

Notably, some studies have been shown that with the use of pneumococcal conjugate vaccination, although there has been a rapid and substantial decline in invasive pneumococcal disease in the target population, there has been an increase in disease due to non-pneumococcal conjugate vaccine serotypes [24,25,26]. In contrast, protein-based vaccines target surface or toxin antigens common across Streptococcus pneumoniae. This design confers broader protection that is not confined to specific serotypes and effectively circumvents the issue of serotype replacement [27]. In comparison with other epidemiological studies, the safety and immunogenicity profiles of the vaccine in this study have certain characteristics. For example, in terms of local adverse reactions, the observed incidence of injection site erythema was consistent with previous clinical finding [28,29], but the overall adverse event and reaction incidences may vary in different studies due to factors such as sample size, study population, and monitoring methods [29]. In terms of immunogenicity, the antibody responses of various serotypes in this study are comparable to those in other similar vaccine studies, but there are also some serotype-specific differences. For instance, a study reported the carriage rate and pathogenic mechanisms of Streptococcus pneumoniae, which is relevant to understanding the background of vaccine prevention [3]. Another study provided information on the antibiotic resistance of the pathogen, emphasizing the importance of vaccination [4]. The limitations of this study are that the study subjects were 12–23-month-old children from specific regions, which may not fully represent the situation of all Chinese children. Moreover, only short-term safety and immunogenicity were observed in this study, and the long-term effects remain to be further investigated. In conclusion, PCV13i has good safety and immunogenicity in 12–23-month-old Chinese children and can provide effective immune protection against pneumococcal infections. Future studies could further expand the sample size and research scope to more comprehensively evaluate the application value of this vaccine in Chinese children.

## 5. Conclusions

Based on the findings of this Phase III clinical trial, the novel PCV13i demonstrates a favorable and acceptable profile for use in 12–23-month-old children. The vaccine exhibited a safety profile comparable to the licensed PCV13, with no vaccine-related serious adverse events reported. Furthermore, PCV13i met the non-inferiority criteria for immunogenicity, inducing robust protective immune responses against the targeted serotypes. In conclusion, PCV13i presents a viable and effective vaccination option for this pediatric age group.

## Figures and Tables

**Table 1:** Baseline of participants.

	Experimental Group (n = 263)	Control Group (n = 263)	*p*
Age (month)	18.11	18.05	0.833
Gender n (%)			0.542
Male	135 (51.33%)	128 (48.67%)	
Female	128 (48.67%)	135 (51.33%)	
Body mass index (kg/m^2^)	11.67	11.58	0.477
Axillary temperature before first dose (°C)	36.46	36.47	0.656

**Table 2:** Adverse reactions within 30 days post-vaccination.

	Experimental Group (n = 520)	Control Group (n = 523)	*p*
Overall adverse events	193 (37.12%)	171 (32.70%)	0.134
Overall adverse reactions	129 (24.81%)	113 (21.61%)	0.221
Systemic adverse reactions	83 (15.96%)	86 (16.44%)	0.833
Local adverse reactions	52 (10.00%)	32 (6.12%)	0.021
Erythema	41 (7.88%)	21 (4.02%)	0.008
Swelling	16 (3.08%)	12 (2.29%)	0.434
Pain	2 (0.38%)	6 (1.15%)	0.291
Fever	56 (10.77%)	72 (13.77%)	0.140
Grade 3	0	1 (0.19%)	>0.999
Irritability	2 (0.38%)	0	0.248
Cough	15 (2.88%)	12 (2.29%)	0.548
Diarrhea	12 (2.31%)	8 (1.53%)	0.360
Vomiting	4 (0.77%)	3 (0.57%)	0.994
Hypersensitivity reaction	1 (0.19%)	0	0.499

“n” represents the total number of participants vaccinated per dose.

**Table 3:** Type-specific seropositive rates and GMC of IgG response before and 30 days post-vaccination.

		Pre-Vaccination				Post-Vaccination			
Serotype	Group	Seropositive Rate% (95%CI)	*p*	GMC (95%CI)	*p*	Seropositive Rate % (95%CI)	*p*	GMC (95%CI)	*p*
1	Experimental group	65.49 (59.30, 71.31)	0.323	0.27 (0.22, 0.32)	0.326	100.00 (98.56, 100.00)	-	3.17 (2.97, 3.38)	<0.001
	Control group	61.26 (54.96, 67.30)		0.23 (0.19, 0.29)		100.00 (98.55, 100.00)		3.89 (3.63, 4.18)	
3	Experimental group	5.49 (3.03, 9.04)	0.451	0.03 (0.03, 0.04)	0.618	96.47 (93.41, 98.37)	0.802	1.12 (1.04, 1.20)	<0.001
	Control group	7.11 (4.27, 11.01)		0.03 (0.02, 0.04)		96.05 (92.85, 98.09)		0.88 (0.81, 0.94)	
4	Experimental group	5.49 (3.03, 9.04)	0.563	0.02 (0.02, 0.02)	0.653	100.00 (98.56, 100.00)	-	3.47 (3.24, 3.72)	0.363
	Control group	6.72 (3.96, 10.54)		0.02 (0.02, 0.03)		100.00 (98.55, 100.00)		3.31 (3.08, 3.57)	
5	Experimental group	59.22 (52.91, 65.30)	0.352	0.24 (0.20, 0.29)	0.716	96.47 (93.41, 98.37)	0.007	0.90 (0.84, 0.96)	<0.001
	Control group	63.24 (56.97, 69.19)		0.25 (0.21, 0.31)		100.00 (98.55, 100.00)		1.94 (1.81, 2.08)	
6A	Experimental group	31.76 (26.10, 37.86)	0.802	0.13 (0.10, 0.16)	0.753	99.22 (97.20, 99.90)	0.499	1.91 (1.75, 2.09)	0.018
	Control group	32.81 (27.06, 38.97)		0.13 (0.11, 0.16)		100.00 (98.55, 100.00)		2.24 (2.03, 2.47)	
6B	Experimental group	80.00 (74.56, 84.73)	0.222	0.44 (0.37, 0.51)	0.380	98.82 (96.60, 99.76)	0.250	2.72 (2.47, 2.99)	0.320
	Control group	75.49 (69.72, 80.66)		0.39 (0.33, 0.47)		100.00 (98.55, 100.00)		2.91 (2.64, 3.21)	
7F	Experimental group	30.59 (24.99, 36.64)	0.361	0.07 (0.05, 0.09)	0.886	100.00 (98.56, 100.00)	-	8.10 (7.52, 8.72)	<0.001
	Control group	34.39 (28.55, 40.59)		0.07 (0.06, 0.08)		100.00 (98.55, 100.00)		6.07 (5.65, 6.51)	
9V	Experimental group	13.33 (9.41, 18.13)	0.616	0.03 (0.02, 0.03)	0.896	100.00 (98.56, 100.00)	-	2.68 (2.47, 2.92)	0.235
	Control group	11.86 (8.15, 16.49)		0.03 (0.02, 0.03)		100.00 (98.55, 100.00)		2.89 (2.64, 3.16)	
14	Experimental group	98.04 (95.48, 99.36)	0.391	1.72 (1.55, 1.90)	0.686	100.00 (98.56, 100.00)	-	10.37 (9.68, 11.11)	<0.001
	Control group	96.84 (93.86, 98.63)		1.78 (1.56, 2.02)		100.00 (98.55, 100.00)		14.24 (13.30, 15.25)	
18C	Experimental group	7.06 (4.24, 10.93)	0.025	0.02 (0.02, 0.02)	0.184	100.00 (98.56, 100.00)	-	2.88 (2.67, 3.11)	0.156
	Control group	13.04 (9.15, 17.83)		0.02 (0.02, 0.03)		100.00 (98.55, 100.00)		3.13 (2.87, 3.41)	
19A	Experimental group	91.76 (87.69, 94.83)	0.620	0.83 (0.71, 0.97)	0.577	100.00 (98.56, 100.00)	-	7.24 (6.73, 7.78)	0.420
	Control group	90.51 (86.21, 93.83)		0.89 (0.75, 1.05)		100.00 (98.55, 100.00)		6.94 (6.43, 7.48)	
19F	Experimental group	92.55 (88.61, 95.45)	0.748	0.85 (0.74, 0.98)	0.768	100.00 (98.56, 100.00)	-	9.98 (9.25, 10.76)	<0.001
	Control group	93.28 (89.46, 96.04)		0.88 (0.75, 1.02)		100.00 (98.55, 100.00)		5.23 (4.85, 5.63)	
23F	Experimental group	41.18 (35.07, 47.49)	0.033	0.18 (0.14, 0.22)	0.581	99.61 (97.83, 99.99)	>0.999	2.65 (2.46, 2.85)	0.076
	Control group	50.59 (44.26, 56.91)		0.19 (0.15, 0.24)		99.60 (97.82, 99.99)		2.38 (2.18, 2.61)	

**Table 4:** Proportion of serum-type-specific pneumococcal IgG antibody concentrations ≥ 1.0 μg/mL.

		Experimental Group (n = 255)		Control Group (n = 253)		
Serotype		n	Proportion % (95%CI)	n	Proportion % (95%CI)	*p*
1	Pre-vaccination	24	9.41 (6.12, 13.68)	24	9.49 (6.17, 13.79)	0.977
	Post-vaccination	248	97.25 (94.43, 98.89)	251	99.21 (97.17, 99.90)	0.182
3	Pre-vaccination	3	1.18 (0.24, 3.40)	3	1.19 (0.25, 3.43)	>0.999
	Post-vaccination	146	57.25 (50.93, 63.41)	96	37.94 (31.94, 44.23)	<0.001
4	Pre-vaccination	2	0.78 (0.10, 2.80)	4	1.58 (0.43, 4.00)	0.674
	Post-vaccination	252	98.82 (96.60, 99.76)	248	98.02 (95.45, 99.36)	0.713
5	Pre-vaccination	10	3.92 (1.90, 7.09)	13	5.14 (2.76, 8.63)	0.510
	Post-vaccination	102	40.00 (33.94, 46.30)	221	87.35 (82.62, 91.19)	<0.001
6A	Pre-vaccination	2	0.78 (0.10, 2.80)	4	1.58 (0.43, 4.00)	0.674
	Post-vaccination	214	83.92 (78.83, 88.21)	213	84.19 (79.10, 88.46)	0.934
6B	Pre-vaccination	46	18.04 (13.52, 23.32)	58	22.92 (17.89, 28.60)	0.172
	Post-vaccination	232	90.98 (86.77, 94.20)	230	90.91 (86.67, 94.15)	0.978
7F	Pre-vaccination	15	5.88 (3.33, 9.52)	12	4.74 (2.47, 8.14)	0.567
	Post-vaccination	255	100.00 (98.56, 100.00)	253	100.00 (98.55, 100.00)	-
9V	Pre-vaccination	2	0.78 (0.10, 2.80)	6	2.37 (0.88, 5.09)	0.280
	Post-vaccination	237	92.94 (89.07, 95.76)	235	92.89 (88.99, 95.73)	0.980
14	Pre-vaccination	221	86.67 (81.87, 90.59)	219	86.56 (81.73, 90.51)	0.972
	Post-vaccination	255	100.00 (98.56, 100.00)	253	100.00 (98.55, 100.00)	-
18C	Pre-vaccination	6	2.35 (0.87, 5.05)	8	3.16 (1.37, 6.14)	0.578
	Post-vaccination	245	96.08 (92.91, 98.10)	241	95.26 (91.86, 97.53)	0.649
19A	Pre-vaccination	128	50.20 (43.89, 56.50)	151	59.68 (53.36, 65.78)	0.032
	Post-vaccination	254	99.61 (97.83, 99.99)	253	100.00 (98.55, 100.00)	>0.999
19F	Pre-vaccination	119	46.67 (40.42, 52.99)	136	53.75 (47.40, 60.02)	0.110
	Post-vaccination	255	100.00 (98.56, 100.00)	253	100.00 (98.55, 100.00)	-
23F	Pre-vaccination	9	3.53 (1.63, 6.59)	9	3.56 (1.64, 6.65)	0.986
	Post-vaccination	243	95.29 (91.92, 97.55)	221	87.35 (82.62, 91.19)	0.001

**Table 5:** GMT and percentage of participants with OPA ≥ 1:8 30 days post-vaccination.

Serotype	Group	GMT (95%CI)	*p*	Percentage % (95%CI)	*p*
1	Experimental group	31.82 (25.11, 40.33)	0.075	91.11 (83.23, 96.08)	0.224
	Control group	43.10 (33.91, 54.77)		95.60 (89.13, 98.79)	
3	Experimental group	185.42 (161.24, 213.24)	0.483	100.00 (95.98, 100.00)	-
	Control group	172.48 (148.57, 200.24)		100.00 (96.03, 100.00)	
4	Experimental group	2631.87 (2165.10, 3199.27)	0.880	100.00 (95.98, 100.00)	-
	Control group	2688.68 (2198.68, 3287.87)		100.00 (96.03, 100.00)	
5	Experimental group	13.43 (9.88, 18.26)	<0.001	74.44 (64.16, 83.06)	<0.001
	Control group	92.95 (66.90, 129.15)		94.51 (87.64, 98.19)	
6A	Experimental group	5980.41 (4536.39, 7884.11)	0.401	98.89 (93.96, 99.97)	0.621
	Control group	4913.92 (3385.37, 7132.63)		96.70 (90.67, 99.31)	
6B	Experimental group	4135.98 (3014.32, 5675.01)	0.238	97.78 (92.20, 99.73)	0.688
	Control group	3046.04 (2032.74, 4564.45)		95.60 (89.13, 98.79)	
7F	Experimental group	39,583.25 (33,086.75, 47,355.31)	<0.001	100.00 (95.98, 100.00)	-
	Control group	17,248.59 (14,213.01, 20,932.50)		100.00 (96.03, 100.00)	
9V	Experimental group	3425.08 (2729.11, 4298.52)	0.594	100.00 (95.98, 100.00)	-
	Control group	3723.45 (3010.18, 4605.73)		100.00 (96.03, 100.00)	
14	Experimental group	3447.71 (2807.82, 4233.41)	0.193	100.00 (95.98, 100.00)	-
	Control group	4138.67 (3433.06, 4989.30)		100.00 (96.03, 100.00)	
18C	Experimental group	1691.44 (1295.27, 2208.78)	0.638	98.89 (93.96, 99.97)	>0.999
	Control group	1849.70 (1417.27, 2414.07)		98.90 (94.03, 99.97)	
19A	Experimental group	3686.14 (3065.41, 4432.56)	0.137	100.00 (95.98, 100.00)	-
	Control group	2995.44 (2438.89, 3678.98)		100.00 (96.03, 100.00)	
19F	Experimental group	1516.83 (1103.50, 2084.97)	0.028	96.67 (90.57, 99.31)	0.605
	Control group	982.95 (787.96, 1226.20)		98.90 (94.03, 99.97)	
23F	Experimental group	8125.45 (6055.05, 10,903.79)	0.290	98.89 (93.96, 99.97)	>0.999
	Control group	6390.47 (4551.71, 8972.02)		97.80 (92.29, 99.73)	

## Data Availability

The data presented in this study are available upon request from the corresponding authors.

## Abbreviations

The following abbreviations are used in this manuscript:

PCV13i	13-valent pneumococcal polysaccharide conjugate vaccine (CRM197/TT)
GMC	Geometric mean concentration
OPA	Opsonophagocytic activity
GMT	Geometric mean titer
IPD	Invasive pneumococcal disease
WHO	World Health Organization
CIES	Carrier-induced epitope suppression
SS	Safety set
BMI	Body mass index
FAS	Full analysis set
PPS1	Per Protocol Set
